# Water and Collagen: A Mystery Yet to Unfold

**DOI:** 10.1021/acs.biomac.4c01735

**Published:** 2025-04-10

**Authors:** Guido Giannetti, Fumiki Matsumura, Federico Caporaletti, Dimitra Micha, Gijsje H. Koenderink, Ioana Mariuca Ilie, Mischa Bonn, Sander Woutersen, Giulia Giubertoni

**Affiliations:** † University of Vienna, Faculty of Physics, Boltzmanngasse 5, 1090 Vienna, Austria; ‡ Max Planck Institute for Polymer Research, 55128 Mainz, Germany; § Laboratory of Polymer and Soft Matter Dynamics, Experimental Soft Matter and Thermal Physics (EST), Université libre de Bruxelles (ULB), Brussels 1050, Belgium; ∥ Amsterdam University Medical Centers (UMC), Vrije Universiteit Amsterdam, 1007 MB Amsterdam, The Netherlands; ⊥ Department of Bionanoscience, Kavli Institute of Nanoscience Delft, Delft University of Technology, 2629 HC Delft, The Netherlands; # Van ’t Hoff Institute for Molecular Sciences, University of Amsterdam, Science Park 904, 1098 XH Amsterdam, The Netherlands; ∇ Van der Waals-Zeeman Institute, Institute of Physics, University of Amsterdam, 1098 XH Amsterdam, The Netherlands

## Abstract

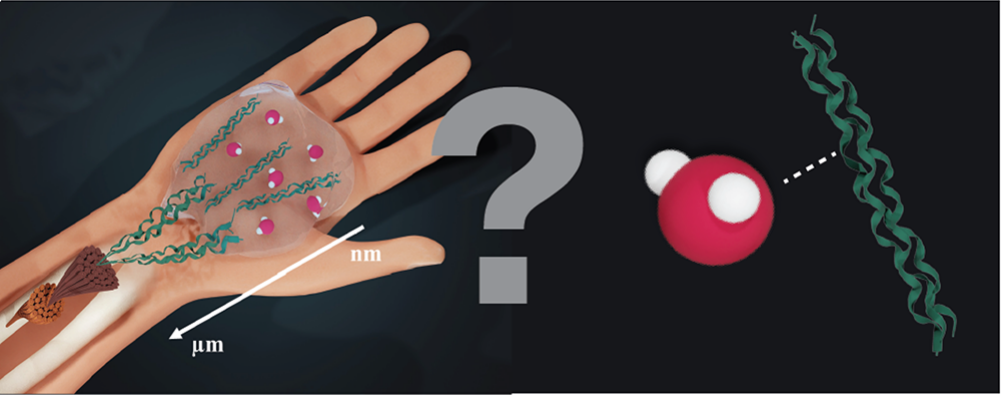

Collagen is the most abundant protein in the human body and plays an essential role in determining the mechanical properties of the tissues. Both as a monomeric protein and in fibrous assemblies, collagen interacts with its surrounding molecules, in particular with water. Interestingly, while it is well established that the interaction with water strongly influences the molecular and mechanical properties of collagen and its assemblies, the underlying mechanisms remain largely unknown. Here, we review the research conducted over the past 30 years on the interplay between water and collagen and its relevance for tissue properties. We discuss the water–collagen interaction on relevant time- and length scales, ranging from the vital role of water in stabilizing the characteristic triple helix structure to the negative impact of dehydration on the mechanical properties of tissues. A better understanding of the water–collagen interaction will help to unravel the effect of mutations and defective collagen production in collagen-related diseases and to pinpoint the key design features required to synthesize collagen-based biomimetic tissues with tailored mechanical properties.

## Introduction

Collagen acts as a scaffold in human connective tissues such as skin, arteries and bones, and imparts these tissues the mechanical properties required to ensure their biological functionality.^1^ Its ubiquitous tissue expression involves production by diverse cell types, including fibroblasts (the main producers), osteoblasts, and odontoblasts. Although no strict consensus exists for the classification of a protein as collagen, 28 different types of collagen have been reported based on the identification of a triple helix conformation, with differences in amino acid type and number.^2^ The most abundant collagen in our body is type I collagen, a fundamental component of connective tissue. Collagen type I is a fibrillar collagen characterized by a single, triple-helical domain. In cells, its biosynthesis starts as a single strand composed of hundreds of amino-acids, the so-called α chain. The type-I collagen chain contains around 1000 amino acids and is composed of Glycine(Gly)-Xaa-Yaa repeat units, where Xaa-Yaa is often Proline (Pro) and Hydroxy-proline (Hyp), respectively. Three α-chains (either two α1(1) and one α2(*I*) chain, or three α2(*I*) chains) wrap around each other adopting a left-handed polyproline II-type (PPII) conformation, and associate to form the typical triple helix, tropocollagen.^3^ The tropocollagens (collagen monomers) are then secretedin the extracellular matrix (ECM), which is a complex and dynamic 3D macromolecular network surrounding the cells. Collagen monomers here self-assemble to form intermediate fibrillar structures (microfibrils) that further associate into fibrils with an ordered molecular packing structure, leading to the formation of sub-band structures in the fibrils, which repeat at a characteristic periodicity (D-band periodicity) of 67 nm.^1^ The importance of this packing process is clearly shown in collagen diseases in which it is perturbed due to defects in the production or structure of collagen type I; as a result, the mechanics of diverse tissue types are affected.^4^ For example, Osteogenesis Imperfecta (Brittle Bone Disease) results in increased bone brittleness, while Ehlers-Danlos syndrome leads to a loss of blood vessel elasticity.

**Figure 1:**
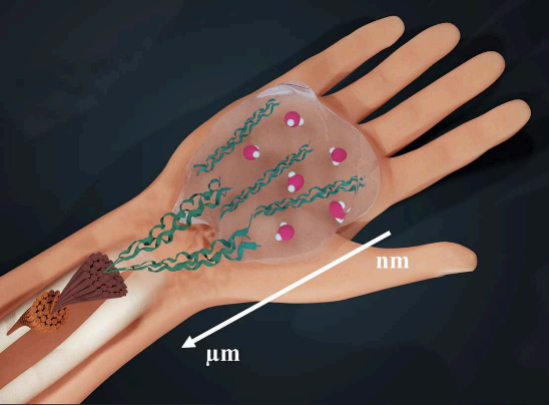
Schematic of collagen assembly. From right to left: tropocollagen molecules interact to form fibrils that then bundle together to form fibers, which act as a scaffold for human tissues such as skin and bone.

Water is one of the principal components in our tissues (ranging from 20 wt % in bones to 80 wt % in cartilage), where it strongly interacts with collagen and plays a major role in defining its molecular and macroscopic properties. For over 60 years,^5^ researchers have been investigating the role of water in determining the molecular and macroscopic properties of collagen. Investigating this topic, however, is challenging because water–collagen interactions occur on many different length and time scales, ranging from single proteins to the full fibrous network (Figure 1). Furthermore, collagen experiences different levels of hydration depending on tissue composition, and has to compete for water with other molecules, such as glycosaminoglycans like hyaluronic acid. The combination of varying hydration and competition with other molecules for water synergistically increases the complexity of water–collagen interactions and their role in defining tissue properties. This complexity is particularly significant when trying to understand how collagen mutations and defects contribute to tissue failure and, ultimately, disease. In this review, we summarize, to the best of our knowledge, the existing literature regarding the interaction between collagen and water on all relevant length scales, from the triple helix up to collagen tissue. We then briefly discuss the potential role of water–collagen interactions in the development of collagen diseases. Finally, we suggest a diverse range of methods to investigate water-collagen interaction and its role in the collagen assembly and its resulting properties. In many respects, water–collagen interaction remains a mystery, and we hope that this review will inspire further research into this fascinating topic.

## Collagen Hydration

The basic structure of collagen in the extracellular matrix is a triple helix, stabilized by interstrand direct hydrogen bonds between the Gly N—H and the C=O groups of the amino acid in position Xaa. Weak hydrogen-bonds between C—H and C=O are also working cooperatively with the N—H···O=C bonds to stabilize the collagen triple helical structure.^7^ Deep-learning simulations together with experimental validation recently have showed that the strength and the number of the hydrogen-bonds determine the stability, helicity and rigidity of the triple helix.^8^ Stability is also partially provided by other weak interactions due to stereoelectronic effects,^9^ as will be discussed later. The collagen structure is such that all Xaa and Yaa residues are well-exposed to the solvent,^10^ leading to specific interactions with the surrounding water molecules.

**Figure 2:**
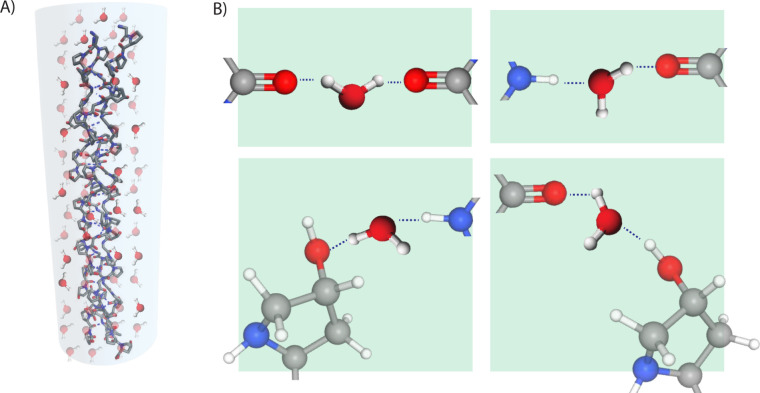
(A) Schematic of collagen hydration. Collagen is shown as a triple helix surrounded by water molecules (in red). (B) Examples of intrachain and interchain water bridges, as proposed in ref (6).

The hydrated structure of tropo-collagen was first studied by Bella et al., mostly using X-ray crystallography.^6,11^ Using collagen-like peptides such as (Pro-Hyp-Gly)_4_-Pro-Hyp-Ala-(Pro-Hyp-Gly)_5_ as models (where Hyp = hydroxyproline), it was shown that collagen is surrounded by “*water molecules in intimate contact with the (collagen) peptide acceptor groups*”, which creates a first hydration shell or cylinder (Figure 2A).^6,11^ These water molecules form a well-organized hydrogen-bonded network, separating the collagen from the bulk solvent and modulating collagen interactions with other molecules (this will be discussed later). Although the definition is not universal, water molecules belonging to the first hydration layer, sometimes referred to as *structural water*, can be further classified into *water-bridges* (water molecules forming direct bridges between polar groups, see below), *cleft water* (water molecules forming chains with tetrahedral angles between the H-bonds in the fiber direction in each groove or cleft of the collagen triple helix), and *interfacial water* (water in direct contact with bulk water).^12,13^ A later nuclear magnetic resonance (NMR) study showed that the first hydration shell is kinetically rather labile, and more exposed to the bulk solvent than in other globular proteins.^14^ The Hyp residues were identified as the “keystone” supporting this water network. Bella et al.^6,11^ suggested that the higher abundance of this particular amino-acid in collagen with respect to other proteins is related to the ability of Hyp to provide hydration sites, while increasing the collagen rigidity by restricting the conformational freedom because of steric hindrance.^6,11^ Further stabilization of this hydration network is likely provided by the carbonyl groups that are not involved in the direct formation of the hydrogen-bonds that stabilize the helix. Simulations showed that the first hydration shell is situated at around 3 Å from the backbone atoms of the collagen, and remains stable at elevated temperature (330 K).^15^ Interestingly, the hydration layer around imino-rich regions (i.e., containing Pro and Hyp) fluctuates less than around imino-poor regions,^15^ again suggesting the importance of Hyp in stabilizing the water network around collagen.

In the core of the first hydration shell, some water molecules form water bridges between polar groups of the triple helix (see Figure 2B for examples from ref (6)). The water bridges can connect polar groups in the same polypeptide strand (intrachain water bridges) or in different α chains (interchain water bridges). Using Molecular Dynamics (MD) simulations, Madhavi et al. showed that at least one water bridge is formed per tripeptide unit in the triple helix.^16^ Anchoring points for the water bridges include hydroxyl groups of Hyp residues and carbonyl groups of Gly and amino acids in the Y position.^6,11,15−17^ Different examples of interchain/intrachain water bridges have been reported in the literature. For instance, MD simulations showed that a stable water bridge is found (although the time-averaged occupancy is ∼ 10%) between the Hyp O—H and Gly C=O on the same chain.^16^ MD simulations performed by Berisio et al.^17^ have suggested that one specific and critical binding site might be also represented by charged arginine (Arg) side chains that, when located in the Y position, are able to form internal water bridges with polar groups in the X+1 positions. Experiments indicate that water bridges in the triple helix exhibit the dynamic and thermodynamic properties of one-dimensional ice.^6,18^ However, other MD simulations showed evidence that these water bridge are not ice-like, despite their insensitivity to temperature and their long residence time (10–100 ps), primarily due to geometrical confinement (rather than a strong enthalpic effect).^16,19^ This discrepancy between experiment and simulation might be due to the intrinsic limitations of MD simulations, for instance, the dependence on specific force-fields. Water bridges are not just present in the triple helix, but also in between triple helixes (so-called interstitial water bridges):^6,15,20,20−22^ when analyzing the crystal structure of collagen-like peptide, Bella et al.^6^ observed that “*the water molecules are organized in a semi-clathrate-like structure that surrounds and interconnects triple helices in the crystal lattice*.” In this case, the amino-acid groups acting as anchoring points for the water network can vary, although MD simulations indicate that charged amino acids are more likely than neutral ones to form water bridges between triple helices.^20^

## Hydration and Its Effect on Collagen Properties

### Hydration and Triple Helix Stability

In this section, we explore the role of water in determining the stability of the triple helix. We will first discuss the contribution of “direct hydration” (first hydration shell and water-bridges) to collagen stability/instability and then the possibility that water might also contribute indirectly to the triple helix stability. Most of the research we present here has focused on investigating this topic by probing the collagen melting temperature (i.e., the temperature at which the triple helix unfolds and the three polypeptide chains adopt a random-coil structure) under different conditions. The melting temperature for a monomeric collagen Type I triple helix in aqueous solution is around 37.5–38 °C, intriguingly close to human body temperature.This temperature’s proximity to the animal body temperature—not only in humans but also in other animals, such as fish, including Antarctic cod^23^—may reflect an evolutionary adaptation, as it facilitates collagen’s dynamic turnover by proteolytic enzymes, which plays an important role in tissue homeostasis and wound healing.^24^

#### Direct Hydration Effects

It has long been hypothesized that the first hydration shell, including specific water-bridges, influences the structure and stability of the triple helix. As already mentioned, the melting temperature of a monomeric triple helix is around 37 °C, but, when incorporated in fibers, the melting temperature of the triple helix increases, dramatically, to 57 °C.^25^ Miles et al.^25^ have suggested that this may be due to the tropo-collagen being spatially confined in a fibril, so its unfolding is inhibited by the loss of configurational entropy of the molecule in the fiber lattice (“polymer-in-a-box” model). Compared to collagen in solution, collagen in a fiber is also partially deprived of solvent, suggesting that dehydration might contribute to collagen stability.^25^ This would be in agreement with the work of Mogilner et al.^26^ who showed using MD simulations that fully dehydrating collagen increases the stability of the triple helix. In this study, however, despite the increased stabilization under dehydrating conditions, the absence of water leads to strong deformation in the helical conformation of the native state, causing the triple helix to bend. This indicates that water might be essential not only for defining thermal stability but also for maintaining the correct functional structure of collagen.^26^

It has also been proposed that collagen hydration could act as a stabilizer because the first hydration shell has to be removed and reorganized to enable the unfolding of the triple-helix. Furthermore, the hydration network provides an appropriate environment for specific water-bridges that might also be crucial to stabilize the collagen structure. The existence of an additional barrier for collagen unfolding was suggested by Gopinath et al.,^27^ who observed that replacing water with ethanol up to 40% leads to a 5 °C lower melting temperature for the collagen triple helix. They suggested that this is caused by “*the disruption of the extensive (water-hydrogen bonded) network surrounding each collagen triple helix*”, lowering the energy barrier for the helix to unwind. In particular, it was suggested that such temperature decrease in the presence of ethanol might be caused by the weakening of water-bridges between specific hydration sites as a result of the disruption of the water network by the ethanol molecules.

The importance of specific binding sites for collagen stability and structure was indirectly revealed by partially replacing water with alcohol. It was found that increasing the number of hydroxyl groups in the alcohol (i.e., increasing the number of groups available for hydrogen bonding) leads to an increase in the stability of the triple helix.^28,29^ This suggested that the cosolvent molecules might anchor to the collagen, creating bridges that further stabilize the triple helix. In particular, Penkova et al.^30,31^ focused on the case of glycerol, showing that this solvent enhances the stability of the triple helix, likely because it replaces water in forming an hydrogen-bonded network around collagen^30,31^ (a similar effect was suggested for ethylene glycol^32^). Specifically, Penkova et al.^30,31^ suggested that the formation of a glycerol bridge between Hyp residues situated in two neighboring triplets of the same strand could play a crucial stabilizing role.

The significance of Hyp for the formation of stabilizing water bridges was also proposed by others.^15,17^ As previously mentioned, MD simulations by Berisio et al.^17^ showed that the rigid side chain of Hyp and also the charged arginine (Arg) side chains, when located in the Y position, are able to form water bridges with polar groups in the X+1 positions of the adjacent strand. Interestingly, the authors examined the stabilizing power of the water-bridges depending on whether these amino acids are present in imino-acid rich or poor regions: in imino-acid poor regions, the Arg water-bridges are not sufficient to stabilize the triple helix, but they might help in the formation of direct hydrogen bonds (i.e., not-water-mediated H-bonds that are formed between the guanidinium group of Arg and the carbonyl moiety of an adjacent chain). Hyp water bridges might instead be relevant in regions of mixed imino-/amino-acid content.^17^ On the other hand, Ravikumar et al.^15^ suggested that water-bridges between Gly C=O and N—H of the amino-acid in position X are critical to ensure the helical stability in imino-poor regions. They also point out that water can play a dual role: if a water-bridge is formed, it will act as a stabilizer, but if water forms a single hydrogen bond to the backbone it will act as a “thermal agitator”, destabilizing the collagen structure.^15^ Such a dual role of water was also presented in recent MD simulations. Adopting a continuum solvation model in the simulations leads to the unwrapping of the collagen helix, leading to more exposed Gly and Hyp C=O groups;^33^ however, when a microsolvation model is used (i.e., a model in which only a few critical water molecules are treated explicitly), collagen adopts a tighter triple-helical structure.

In conclusion, the various studies we referenced indicate that water affects collagen stability in two distinct ways: while the hydration network may destabilize the structure in some aspects, it also facilitates the formation of water bridges at specific binding sites, which could be crucial for the stability of the triple-helical structure.

#### Indirect Hydration Effects

**Figure 3:**
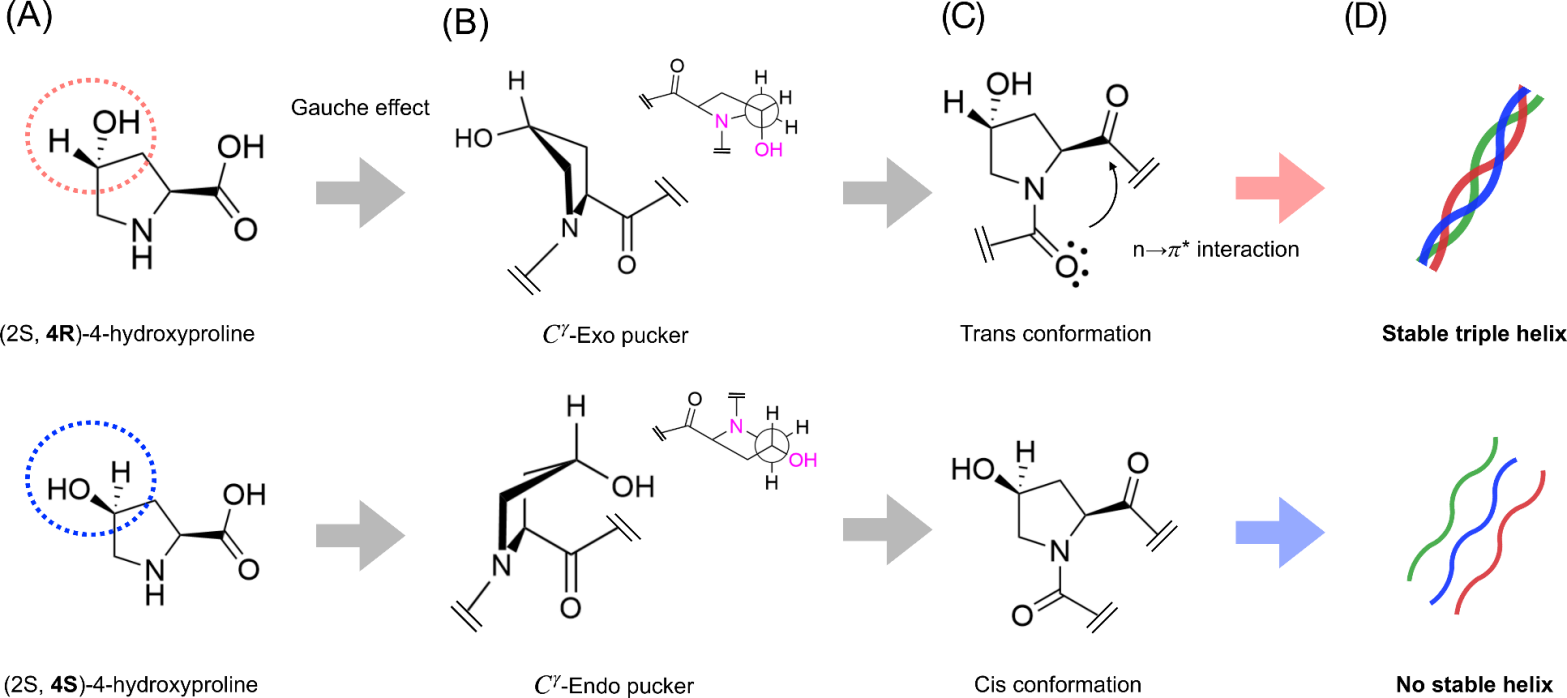
(A) The chemical structures of the (2*S*,4*R*)-4-hydroxyproline and its diastereomer, (2*S*,4*S*)-4-hydroxyproline. (B) The structures of the gauche (*C*^γ^-Exo pucker) and anti (*C*^γ^-Endo pucker) conformers of the pyrrolidine ring. (C) Trans and cis conformers of the adjacent carbonyl groups. The donation of the electron lone pair from the amide oxygen (O_*i*–1_) to the subsequent carbonyl group (O_*i*_) in the *n* → π* interaction is shown as an arrow. (D) Resulting triple helix stability of a collagen-modeled peptide (ProHypGly)_10_ with 4*R* and 4*S* conformers of the Hyp residue.^34^

The role of hydration in the stabilization (or destabilization) of the collagen triple helix might also be indirect. There has been a long controversy about whether the role of imino acid residues (Pro and Hyp) as stabilizers is due to hydration or to stereoelectronic effects, in particuar the *n* → π* interaction between the O and C atoms of the backbone carbonyl groups on either side of a Pro or Hyp residue (Figure 3C).^35,36^ In addition, also if the stability is provided by the latter, it is still debated whether water–collagen interactions might compete with stereoelectronic ones, influencing the strength of *n* → π* interaction and thus the collagen stability.

Raines and co-workers have extensively investigated the stereoelectric effects on the stability of the triple-helical structure. They observed an increase in the melting temperature of the triple helix upon replacing the Hyp residues in the model peptide (ProHypGly)_10_ by fluoroproline (Flp), which forms fewer hydrogen bonds than Hyp does.^9^ Modification of the hydroxyl group of the hydroxyproline to a methoxy group (—OH to —OCH_3_) also increases the melting temperature.^37^ This suggests that not only water bridging but also stereoelectronic effects play a role. Interestingly, replacing (2*S*,4*R*)-4-hydroxyproline with its diastereomer, (2*S*,4*S*)-4-hydroxyproline, dramatically alters the stability of the triple helix. The difference between these two diastereomers is the orientation of the OH group on the fourth carbon of the pyrrolidine ring (see Figure 3A). This substitution strongly reduces the melting temperature, from 60 °C to a point where the helix is unstable even at room temperature.^34,38^ This drastic change has been explained by two stereoelectronic effects, namely, the gauche effect and the *n* → π* interaction. The gauche effect (see Figure 3) is the preference of the gauche conformation (*C*^γ^-exo pucker) over the anticonformation (*C*^γ^-endo pucker) when an electron-withdrawing group is placed at 4*R* position of the pyrrolidine ring. The *n* → π* interaction consists of the donation of the electron lone pair on the amide oxygen (O_*i*–1_) to the empty π* orbital of the subsequent carbonyl group (C_*i*_=O_*i*_). When the hydroxyl group is placed at the fourth carbon of the pyrrolidine ring in the 4*R* conformation, it will stabilize the gauche conformation (*C*^γ^-exo pucker) due to the gauche effect. Adopting a gauche conformation (*C*^γ^-exo pucker) leads to the preference of the trans conformation of the adjacent carbonyl groups, which is stabilized by the *n* → π* interaction.^39^ Collectively, these stereoelectric effects organize the dihedral angles of the main chain, promoting a stable triple helix structure. In addition to these effects, hydration is still a significant factor in stabilization of the collagen. A thermodynamic study by Nishi et al. demonstrated that the collagen peptide (ProHypGly)_10_ is stabilized by enthalpy via increased hydration, while (ProFlpGly)_10_ is stabilized by entropy via disrupted hydration.^40^ In aqueous solution, the *n* → π* interaction competes with hydrogen bond interactions, likely leading to the destabilization of the triple helix.^41^

### Hydration and Collagen Self-Assembly

As discussed, collagen is surrounded by a tight hydrogen-bonded network of water molecules, which plays an important role in defining the properties of the triple helix. Yet, the influence of water extends beyond the level of the triple helix and also impacts collagen properties at the fibrous-network level, primarily by directing and guiding the collagen assembly process.

Water assumes different roles during this complex process (which must proceed correctly in order to guarantee the functionality of tissues). For instance, it is believed that the first hydration shell defines the boundary between the collagen monomers during assembly. This implies that as two monomers approach each other they interact in such a way that the void regions within their hydration shells overlap. This interaction establishes a minimum distance between the monomers, governed by the stability of the hydration shells, which contributes to the precise interaxial spacing observed in collagen assemblies.^15^ Water would thus act as “*lubrication layer*” that assists during the assembly by promoting the specific coordination between the collagen monomers, as elegantly stated in ref (42). Based on measurements of collagen assembly in the presence of sugars and polyols, it was proposed that more specific “hydrogen-bonded water clusters bridging recognition sites on the opposing helices” are required for fibrillogenesis.^21^ Kuznetsova et al.^21^ have suggested that the ability of sugars and polyols to inhibit collagen assembly is related to their ability to compete with water to hydrogen-bond with collagen, disturbing its hydration sites, and thus the recognition process. Although glycerol experiments indicate that the hydration sites surrounding Hyp are the most critical, it remains unclear which specific hydration sites are the main determinants of the recognition process.

Interestingly, it has been shown that if the hydration is reduced, for instance by adding ethanol^27^ to the solvent, the assembly occurs faster. This indicates that a crucial stage in collagen assembly involves the removal of water from the collagen surface, a phenomenon also observed in a recent molecular MD study.^43^ The idea that the partial depletion of water is important for the assembly has also been proposed to explain the increase in assembly rate when raising the temperature: at higher temperature, the water is less strongly bound to the collagen surface, favoring hydrophilic interactions between the monomers.^22,44^

**Figure 4:**
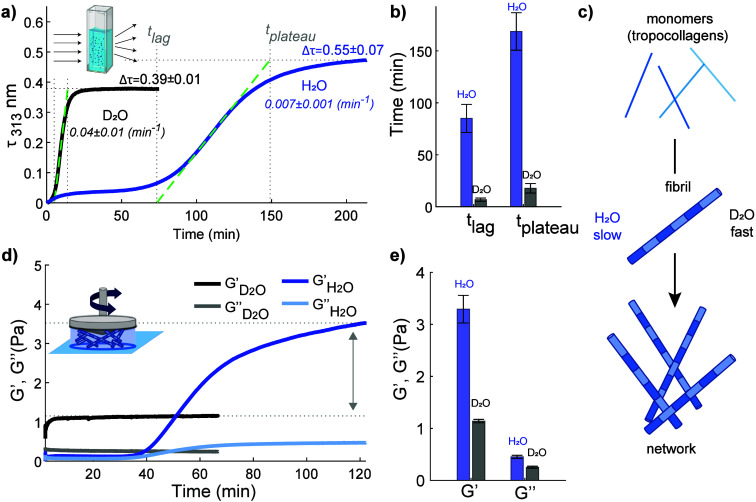
Differences in collagen assembly kinetics and collagen-network elastic properties in H_2_O and D_2_O. (a) Turbidity measurements for water and heavy water solutions containing Type I full-length collagen at a concentration of 0.1 mg/mL measured at a temperature of 23 °C. Spectra were collected every 15 and 30 s for D_2_O and H_2_O experiments, respectively. (b) Lag and plateau time values found for collagen fibrilization in H_2_O and D_2_O. (c) Schematic of collagen assembly in water and heavy water. (d) Rheology measurement for water and heavy water solutions containing collagen at a concentration of 0.5 mg/mL. Measurements were conducted at a strain amplitude of 0.8%, an oscillation frequency of 0.5 Hz and temperature of 23 °C. (e) Elastic and viscous moduli after attaining the plateau level. Figure adapted from ref (45) by Giubertoni et al. This work is licensed under a Creative Commons Attribution 4.0 International License (CC BY 4.0).

In our own lab, we recently investigated the effect of replacing water by heavy water on the collagen assembly.^45^ Although heavy water (or D_2_O) has the same size and dielectric constant as water,^46^ collagen fibrillogenesis happens 10 times faster in D_2_O than in H_2_O (Figure 4). We believe that this is because collagen in heavy water has a less solvent-exposed structure (as has been observed previously for other proteins^46^); as a consequence, the energetic cost to remove water from the collagen surface (desolvation energy) is lower, and the assembly occurs faster in D_2_O. These results again demonstrate that water is not a passive bystander during collagen assembly, but mediates, directs and guides collagen fibrillogenesis.

### Hydration and Collagen Fibrils

The structure and interactions of collagen triple-helix monomers inside fibrils are also strongly influenced by water, leading to an hydration-dependence of the mechanical properties of the fibrous network and of the tissues. The water inside fibrils forms a complex water-bridge network that interconnects the different helices.^6^ In ref (20), it was shown using MD simulations that the density of these water-bridges increases in the overlap regions of collagen (i.e., the regions where there is a clustering of charge–charge interactions). This hydration network is fundamental for the stability of the fibrils. Leikin et al.^47^ demonstrated with Raman spectroscopy that rearrangements of the complex hydration network in the fibrils causes an interhelical force (‘hydration force”) which is attractive at long (≥15 Å) distance and repulsive at short (≤15 Å) distance. This idea was already suggested previously by the same authors based on osmotic-stress results.^22^

**Figure 5:**
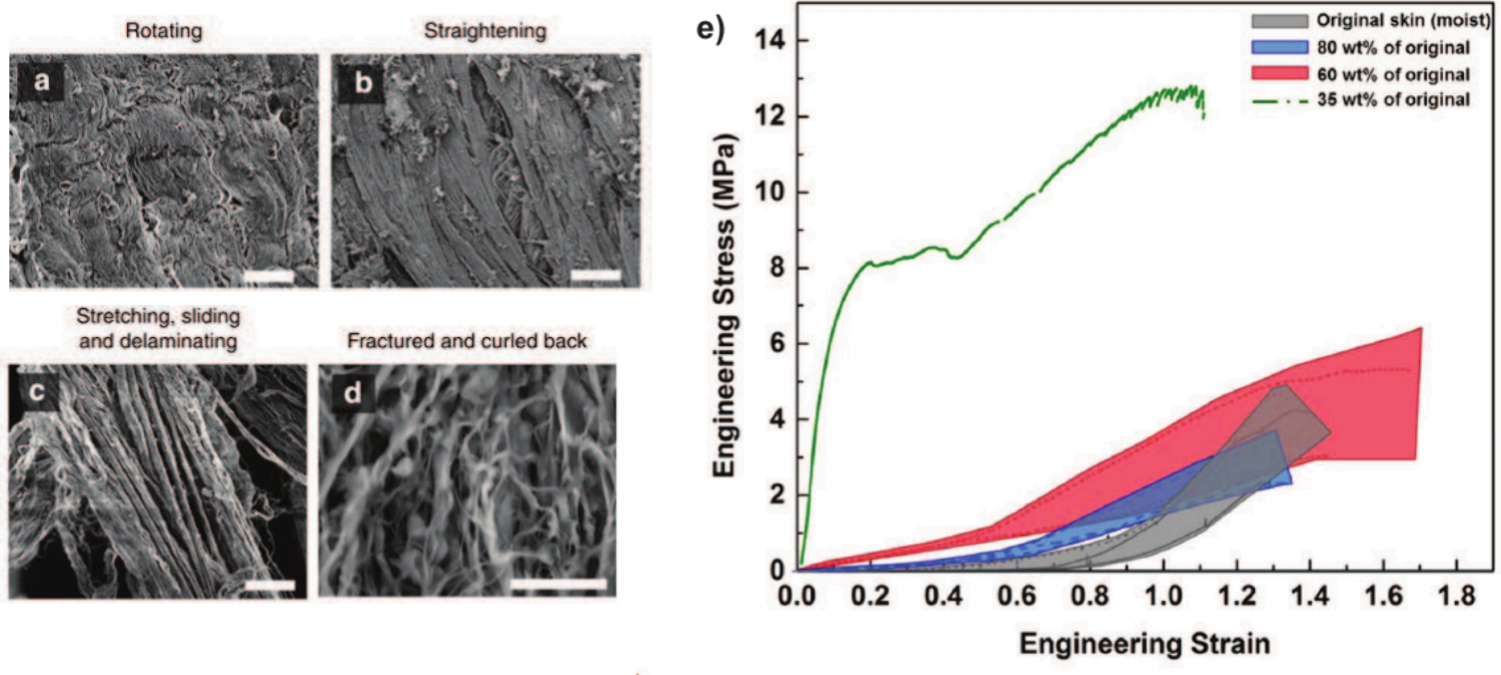
SEM images (a–d) of the mechanisms during the four stages of tensile loading of rabbit skin. (a) Curved collagen fibrils are oriented along the tensile axis; (b) collagen fibrils are straightening, increasingly large amounts of the fibrils reorient close to the tensile axis; (c) collagen fibrils are stretching, sliding, delaminating, and are orientated completely along the tensile axis; (d) collagen fibrils are fractured and curled back. Scale bars in (a–d) are 20, 20, 20, and 50 μm, respectively. (e) Stress–strain scatterband curves of original moist skin and different dehydrated skin (80, 60, and 35 wt % of moist skin). As skin becomes dehydrated, the toe region of the stress–strain curves becomes shorter and stiffer. After losing 65% weight due to dehydration, the toe region of the stress–strain curve has completely vanished. Figure adapted from ref (48) by Yang et al. This work is licensed under a Creative Commons Attribution 4.0 International License (CC BY 4.0).

Water molecules inside the fibrils also have an important role in regulating the mechanical response of the fibrils to external deformation, as observed in experiments, where the mechanical response of tissues was measured at different degrees of hydration. Yang et al.^48^ imaged the reorganization and rearrangement of the fibril network while applying mechanical deformation to skin samples obtained from rabbits (see Figure 5a–d). Stress–strain curves of skin obtained at different hydration levels show an increase of stiffness upon dehydration^48^ (see Figure 5e); similar mechanical response was observed for tendons and other tissues.^49−54^ Such a stiffening response has also been observed in isolated collagen fibrils.^55−60^ Interestingly, in ref (56), shrinking and stiffening of collagen fibrils were observed when decreasing the water content in the fibrils while increasing the osmotic pressure by using molecular crowding agents, such as polyethylene oxide (more commonly known as PEG or PEO). In bones, it has been shown that dehydration leads to the reorientation of the fibrils (and in this way to a mechanical response).^61^

The mechanism by which water regulates the macroscopic mechanical response of the collagen network (and of tissue) has been extensively studied. Gautieri et al.^59^ showed using simulations that at low hydration level collagen molecules slide past each other by a stick–slip mechanism which involves the breaking of the direct hydrogen bonds between the neighboring helices. When hydrated, water bridges replace the hydrogen bonds, smoothing the movement of the helices by reducing the stick–slip sliding.^51,59^ Water is thus crucial for transferring the mechanical load between the tropocollagens, and for acting as a “lubricant” to facilitate the sliding of the helices during mechanical deformation, as shown in Figure 5c.^48,59,62,63^ A similar explanation was proposed by Bhattacharya et al.,^64^ who investigated the role of water in the mechanical properties of collagen Type I and Type II using MD simulations. They proposed that the presence of interstitial water weakens the interhelix interactions, reducing the tensile modulus and softening the fibrous network. It has also been suggested that the role of water depends on the applied deformation, acting as a lubricant in the case of axial stretching, but as glue during axial sliding and microfibril bending.^10^ In the latter case, water can act as a glue because, in order to move the monomers apart, water bridges have to be broken and reorganized.^10^ Through MD simulations, by varying the hydration level in a microfibril crystal, Vassaux^65^ identified a specific hydration range (between 90%–100%), where both electrostatic and van der Waals interactions increase. This range is suggested to be optimal for force transfer, triggering the glue-to-lubricant transition. Notably, this hydration level is higher than the physiological level measured in rat tail fibers (∼62%), which may indicate that collagen fibrils are not solely designed to draw optimal mechanical potential from hydrogen bonds.

An interesting study by Masic et al.^50^ has shown that water removal changes the conformation of collagen, leading to a shortening of the molecule (by ∼ 1.3%), and creating high tensile stress. A mechanical response already occurs when the osmotic pressure surrounding the collagen experiences a slight change, similar to the one exerted by other components of the tendon such as proteoglycans. The shrinkage of the collagen fibrils in dehydrating conditions was also suggested in ref (66), where it was proposed that the dehydration process happens in two steps: in the first step, the lateral spacing between the collagen molecules is reduced, and in a second step the collagen molecules shorten. The authors of ref (50) conclude that “*much like steel fibers in armoured concrete, collagen that shrinks axially during mineralization would put compressive load on the rest of the structure, protecting the mineral phase from tensile loads.*” The role of water thus seems to be not simply to lubricate and facilitate the movement of the collagen molecules but also to actively control the tension of the fibril by adjusting the contour length of the collagen molecule.

### The Effect of Hydration on the Interaction of Collagen with Other Molecules: The Case of Bone Tissue

**Figure 6:**
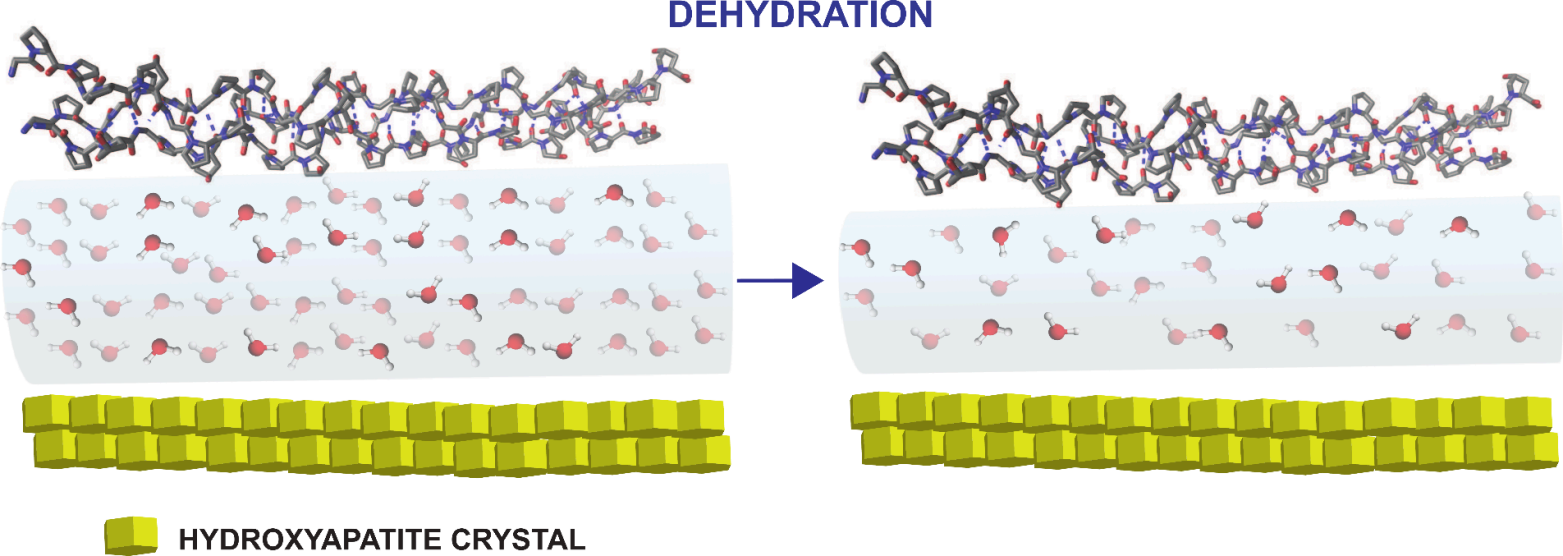
Model showing the effect of dehydration at the collagen/inorganic (hydroxypatite) interface in bone tissue. After dehydration, the distance between the collagen and inorganic surface is reduced. This figure is based on ref (69). Copyright 2011 American Chemical Society.

It has been long recognized that water not only guides collagen–collagen interactions but also acts as an “interfacial agent” to mediate collagen interactions with other molecules. For instance, various studies have explored into the significance of water in mediating collagen interactions with apatite crystals,^67−71^ a key component of human bones together with water and collagen. For a comprehensive understanding of this topic, we recommend referring to a recent review by Surowiec.^70^ In a study by Wilson,^67^ the existence of a water layer between bone crystallites and collagen was shown by using nuclear magnetic resonance (NMR). A later NMR study showed that this water layer is a strongly hydrogen-bonded network,^72^ and its presence and organization are evidenced by the observed reduction in the distance between the collagen and the inorganic regions upon dehydration, or upon replacing H_2_O with D_2_O (Figure 6).^69^ The existence of a water layer between collagen and the inorganic surface might be crucial because it prevents the formation of direct cross-links of the hydroxypatite with collagen.^69^ Such direct cross-links have been associated with bone weakening, and observed to increase with aging.^69^ Recent work has shown that water plays an important role in defining the mechanical properties of mineralized fibrillar collagen, by softening the material and by increasing its capability of dissipating mechanical energy due to applied loading.^73^ It has been suggested^69^ that the interfacial layer acts as a lubricant between collagen and minerals, enabling bones to sustain external stress (similarly to what was suggested for the water layer between collagen fibrils), and that this hydrogen-bonded network could act as a “*a sacrificial layer, protecting collagen from shear under uniaxial stress*”. At early stages of mineral formation, the minerals are also thought to affect collagen hydration, probably leading to structural changes.^71^

The above results show how water influences the interaction between collagen and other bone components. We focused on the case of bone tissue, since it has been studied much more extensively than other tissues and provides the best illustration of water’s function as a mediator between collagen and its surrounding environment. Given that collagen is generally not in a very hydrating environment (water comprising only 20 wt % of bone tissue and 80 wt % of cartilage, the most hydrated tissue in our body) and faces significant competition from highly hydrophilic molecules, such as glycominoglycans, it is likely that the hydration shell enveloping collagen serves as a mediator between collagen and other molecules also in other tissues besides bones.

## Water and Collagen: A Missing Piece of the Puzzle to Determine the Criteria for Tissue Failure?

### Advanced Glycation End-product (AGE) Diseases

Dysfunction of collagen tissues due to Advanced Glycation End-products (AGEs) is one of the most common causes of age-related collagen diseases.^74^ The enzymatic reactions that covalently bind sugar groups to collagen type I are enhanced with aging, leading to altered mechanical responses in collagen tissue, including stiffening, increased failure load, decreased viscoelasticity in tendons, and loss of bone plasticity and toughness, particularly in the elderly and those affected by Type-II diabetes.^74−76^ Computational modeling by Kamml et al.^77^ and experimental studies by Gautieri et al.^76^ have shown that at the nanoscale, AGEs formed between triple helices act as sliding inhibitors (contrary to water which acts as a lubricant). This inhibition results in fibrillar deformation being dominated by the deformation of collagen molecules, leading to D-banding loss and abrupt failure.

Despite the significance of AGEs, many questions remain about the molecular mechanisms underlying the onset of pathological conditions. One key question is whether potential alterations in hydration might be present and consequently exacerbate the effect of AGEs on collagen-related diseases. In vitro studies by Andriotis et al.^78^ on collagen fibrils with increased glycation revealed heightened hydration and reduced stiffness compared to untreated fibrils. This suggests a connection between AGEs and altered hydration. Nash et al.^79^ investigated the effects of glucosepane on collagen fibrils, which is the most abundant AGE that forms intramolecular cross-links (i.e., formed within the same triple helix). They observed an increase in the Surface Accessible Area (SASA) following the insertion of a single cross-link. This increase, attributed to greater interchain spacing within the triple helix, facilitated solvent accessibility and water diffusion around the collagen backbone.

Interestingly, ex vivo experiments reported by Trębacz et al.^80^ pointed to a decrease (rather than an increase) of hydration. In this work, the authours showed that in naturally aged rabbit tissues the denaturation temperature increases due to a greater number of cross-links, in a manner similar to in vitro experiments. However, they observed a decreased in the enthalpy of denaturation (Δ*H*) suggesting either or a reduced hydration, in contrast to what is observed in vitro, or tighter fibril packing over time. On the other hand, measurements performed by Nash et al.^79^ on human tendons revealed an increase in free water content in older donors (71.2 ± 6.0 years, *n* = 6) compared to younger donors (16.7 ± 2.7 years, *n* = 6).

Although these findings clearly show that AGE formation—whether under short-term in vitro ribosylation or long-term in vivo conditions—always alters collagen hydration, a systematic understanding of the effect of AGEs on collagen–water interactions and its potential consequences for the mechanical behavior of AGE-rich collagen tissues is still lacking.

### Genetic Collagen Diseases

Mutations in collagen genes lead to changes in collagen production and/or sequence, leading in turn to defects in fibrillar collagen α chains and thus to tissue dysfunction. The clinical presentation of the associated diseases is quite variable: disease manifestations range from limited tissue types to the presentation of a generalized multisystemic character, which may depend on the tissue expression pattern of the collagen type, as well as the genetic defect (Table 1). Alteration of the collagen hydration in these diseases has been investigated mostly at the level of the triple helix. Mutations in the collagen sequence, such as for instance replacement of Gly with Ala, were shown to increase the bound water to the collagen by replacing the interchain hydrogen bonds in the triple helix with water bridges, which are known to be weaker and thus lead to the destabilization of the triple helix.^6,11,81,82^

**Table 1:** Human Monogenic Diseases Associated with Fibrillar Collagen Dysfunction

Fibrillar collagen gene	Disease	Primary affected tissue
COL1A1	Osteogenesis imperfecta	Bone
	Caffey disease	Bone
	Ehlers Danlos Syndrome	Skin, arterial
COL1A2	Osteogenesis imperfecta	Bone
	Ehlers Danlos Syndrome	Skin, cardiac
COL2A1	Achondrogenesis	Cartilage
	Spondyloepiphyseal dysplasia	Bone
	Legg-Calve-Perthes disease	Vascular
	Kniest dysplasia	Cartilage
	Stickler syndrome, type I	Vitreous, retina
COL3A1	Ehlers Danlos syndrome	Arterial
COL5A1	Ehlers Danlos syndrome	Skin
COL5A2	Ehlers Danlos syndrome	Skin
COL11A1	Marshall syndrome	Eyes, joints
	Stickler syndrome, type II	Ocular
COL24A1	None	N/a
COL27A1	Steel syndrome	Bone

In contrast to the effect on the monomeric triple helix, the effect of the modified water–collagen interaction on the final clinical presentation has so far been mostly neglected. For many years, indeed, the clinical picture has been investigated only in relation to the function of the cells residing in these tissues and their quality as a direct result of collagen dysregulation. Despite the increasing awareness of the impact of hydration on collagen properties,^45^ this knowledge has not been yet applied in the study of relevant genetic diseases. Limited studies have been conducted on osteogenesis imperfecta (OI), a genetic disorder of bone fragility due to defective collagen type I. Fourier transform infrared spectroscopy has been used to investigate bone tissue composition in homozygous oim mice, an OI mouse model only producing COL1A1 homotrimers due to the genetic disruption of *Col1a2*.^83^ The oim mice demonstrated lower protein and higher water content compared to their wild-type counterparts, which was attributed to more matrix space in oim mice, which can facilitate loosely bound water; this effect correlated with compromised mechanical properties.^84^ In agreement with these findings, another study found that oim collagen type I is more loosely packed and it has more bound water and nonenzymatic cross-links, AGEs. On the other hand, hydration resulted in less unbound water, possibly due to the increased bound water.^85^ Critical information is missing regarding insights into hydration in other tissue types affected by collagen diseases.

In summary, the reported literature indicates a correlation and possibly causal relation between disease and water interaction. Despite this, the potential role of water in the clinical presentation of these diseases has been largely overlooked. We hope this review will inspire further interdisciplinary research into collagen–water interactions and their role in collagen diseases, aiming for a deeper understanding of these interactions that could lead to better targeting and treatment.

### Multiscale and Interdisciplinary Approaches to Unfold the Water–Collagen Mystery

**Figure 7:**
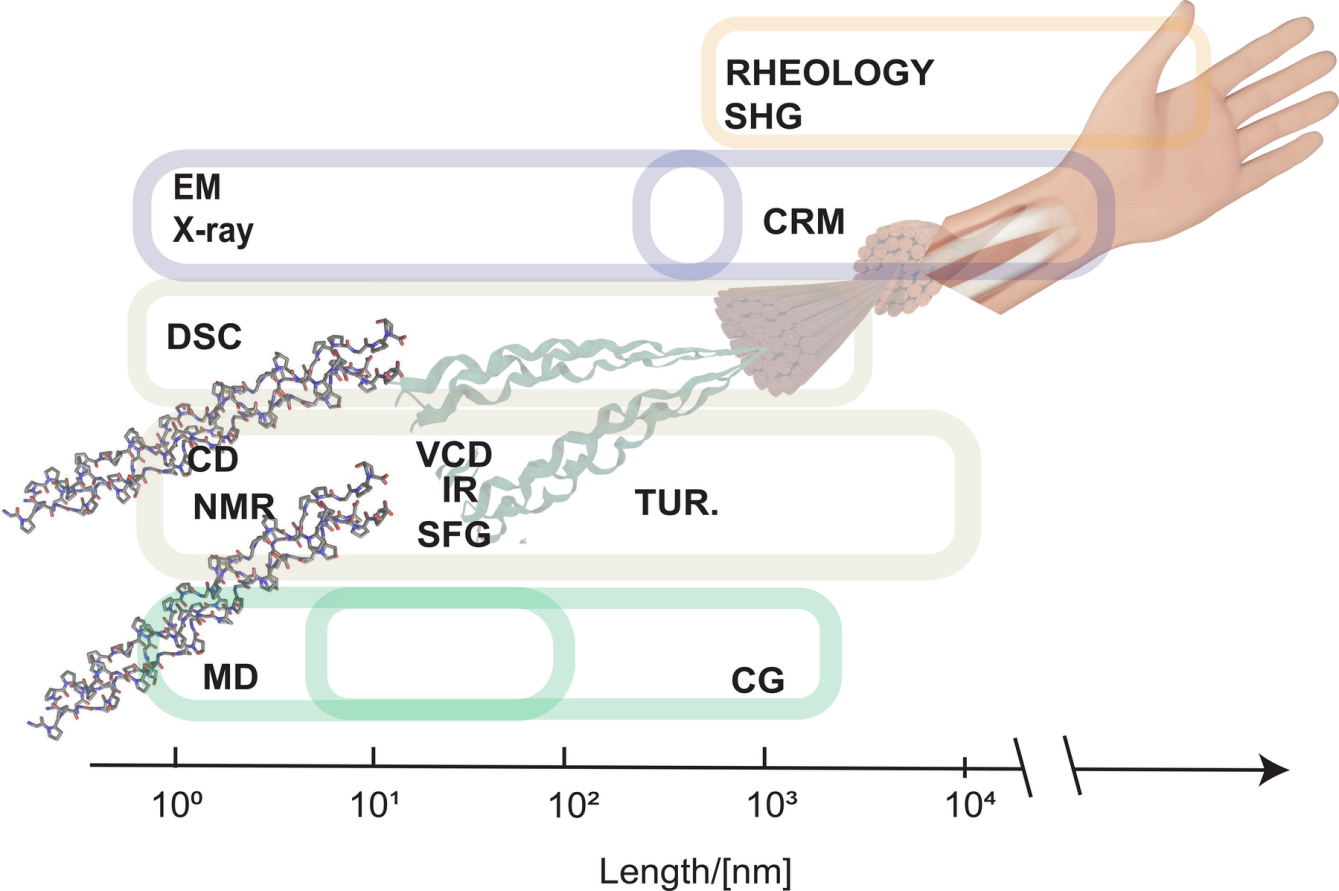
Schematic representation of the spatiotemporal scales of simulation protocols and experimental methods. MD, molecular dynamics; CG, coarse-grained simulations; NMR, nuclear magnetic resonance spectroscopy; IR, infrared spectroscopy; SFG, Sum Frequency Generation spectroscopy; CD, circular dichroism; VCD, vibrational CD; Tur., turbidity; SHG, second-harmonic generation; CRM, confocal reflectance microscopy; EM, electron microscopy; X-ray, protein crystallography.

Interdisciplinary and multiscale approaches are necessary to obtain a comprehensive understanding of the role of water in determining the structural functionality (or dysfunctionality) of collagen. Different techniques are required to investigate the full range of relevant length and time scales, all the way from the molecular level up to macroscopic structure and dynamics. We will give a broad overview of the computational and experimental methods that can be used for addressing the different research questions that we just discussed (Figure 7). Researchers should ensure that in vitro studies are conducted at well-defined and biologically relevant hydration levels to closely mimic the natural hydration state of collagen. We want to emphasize that elucidating the role of water in determining collagen properties requires not only a multimethod approach but also well-defined model systems. Typically, full-length collagen research is based on collagen extracted from animal tissues, which often results in variability because of contaminants, such as cross-linked products. In the future, it would be interesting to use collagen derived from mammalian cells, transgenic plant cells, or genetically modified microbes.^86,87^ We believe that using such well-defined model systems in combination with the set of techniques that we discuss below could be extremely powerful in uncovering the mysterious relationship between water and collagen.

#### How Can We Investigate the Effect of Water on Triple-Helix Structure and Stability?

Various experimental and computational techniques can be used to explore how water impacts the structure and stability of the triple-helix. Because of the significant developments in X-ray technology, X-ray methods have become some of the most effective techniques for studying how changes in hydration influence the structure of collagen, particularly through X-ray scattering, which is an invaluable tool for obtaining structural information on macromolecular structures^88^ such as collagen.^11,89^ Recent advancements in X-ray sources and the advent of X-ray free electron lasers (XFEL) are paving the way to structural 3D-studies of biomacromolecules with unprecedented temporal (≃100 fs) and spatial resolution (≃Å).^90−92^ The much higher brightness of XFELs as compared to conventional synchrotron radiation facilities allows recording diffraction patterns with a single X-ray pulse, i.e., before radiation damage takes place (“diffraction before destruction”^93−95^), removing the need for cryogenic cooling.^92^ Structural information can therefore be achieved even at room temperature, reducing the constraints imposed by crystal size or quality or even entirely removing the need for crystallized samples. This allows studying macromolecules in physiological conditions^92^ and obtained unbiased structural information. Recent developments are leading to the study of the conformation of complex biomacromolecules in aqueous environments^92^ and even in pump–probe experiments, enabling the study of induced structural transitions, as recently demonstrated, for example, in the case of Rhodopsin.^96^

**Figure 8:**
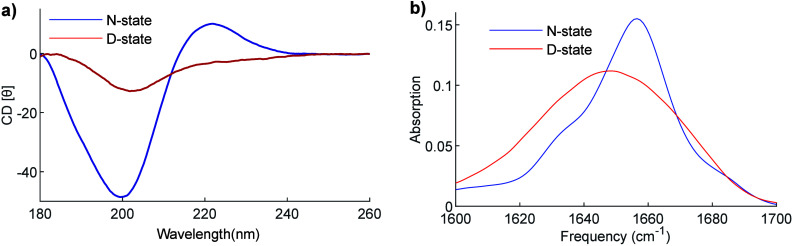
(a) CD spectra of the native (N) and denatured state (D) of type I collagen. Figure adapted from ref (45) by Giubertoni et al. This work is licensed under a Creative Commons Attribution 4.0 International License (CC BY 4.0). (b) IR spectra of the N- and D-state of type I collagen (unpublished data). CD and IR spectra for the N-state are measured at room temperature, while for the D-state, at 60 °C.

X-ray experiments capable of investigating collagen structure in aqueous solutions can nicely complement linear and multidimensional infrared (IR) and circular dichroism measurements, both of which are spectroscopy methods used to study molecular structures in solution. The frequency, width and intensity of absorption peaks in the infrared region caused by the molecular vibrations in the amide groups are highly sensitive to structural and environmental changes.^45,97^ IR-based methods offer many advantages, but they generally require high (unphysiological) concentrations of collagen (∼2–10 mg/mL), and often also the use of D_2_O as a solvent instead of water (to avoid spectral congestion), which might lead to differences in the network structure.^46^ Circular dichroism (CD) is instead particularly sensitive to the helicity of the triple helix,^8,82^ because collagen shows a specific CD signal that arises by its helical structure (see Figure 8a). Contrary to IR, CD can be done at relatively low concentrations (∼0.1 mg/mL) and in water. Both IR spectroscopy and CD can be used also to study the thermal stability of the collagen, by monitoring the change in the IR and CD spectra as a function of temperature (see Figure 8).

Differential Scanning Calorimetry (DSC) is an effective and standard method to assess the thermal stability of collagen. However, standard DSC usually requires large masses to operate (≃100 mg), which makes it unsuitable for in clinical studies, where only small quantities of biological samples might be available. Ultrafast chip calorimetry can circumvent this problem by applying high heating and cooling rates (up to 1 × 10^4^ K/s). The advantages are twofold: thanks to the high temperatures rates achievable, (i) samples with masses as small as 20 ng can be investigated and (ii) high temperature thermodynamic properties (e.g., glass-transition and melting temperatures), water content and fast structural modification can be accessed before degradation takes place, as already demonstrated for other macromolecules.^98−100^ From a computational perspective, various techniques have been employed to investigate the effect of water on the structure and stability of the collagen triple helix. Quantum mechanical (QM) methods are instrumental in capturing the electronic properties of molecules, aiding in the study of charge distributions, proton transfer, and reaction mechanisms. Density Functional Theory (DFT) has been employed to study the effects of different puckering conformations of the pyrrolidine ring, both in vacuum^101^ and aqueous environments,^102^ using Polarizable Continuum Models (PCM) to simulate bulk water properties.^103^ This approach enables the investigation of how water might influence *n* → π* interactions in collagen.^104,105^ DFT has also been employed to predict IR spectra in both vacuum and explicit microsolvation environments, aiding in the interpretation of complex spectra with a focus on solvent effects.^106^ Similarly, chemical shifts for targeted amino acids can be predicted, which, when combined with NMR experiments, provide insights into the interaction between hydration water and the collagen backbone.^107,108^ While DFT provides detailed insights into electronic structure and chemical interactions, its high computational cost, even in vacuum, limits its application to short collagen triplets.^109^ Incorporating solvent effects through methods like microsolvation and PCM—capturing explicit hydrogen bonds and bulk solvation—further increases the computational demand, making large-scale simulations of collagen–water interactions impractical. Additionally, to accurately mimic the collagen environment, dynamic effects must be included as they provide insights into time-dependent phenomena such as folding and stability. Within the quantum mechanical framework, these can be rigorously accounted for using Ab Initio Molecular Dynamics (AIMD) with explicit solvent.^110^ Unfortunately, this approach is currently limited due to the large size of collagen models.^111^ An alternative is to rely on atomistic simulations, which implicitly account for electronic degrees of freedom, reducing computational time and enabling microsecond-scale analyses. This method offers detailed insights into the structural and dynamic properties of collagen in different solvents, aiding the exploration of water’s role in collagen stability.^112,113^

Given the complexity and heterogeneity of native collagen, simulations of collagen model peptides (CMPs) composed of Pro-Hyp-Gly repeats have been essential in uncovering the structure–property relationships in collagen triple helices. Recent thermal denaturation simulations in explicit water have explored various CMP models, including constitutional isomers,^114^ capped and uncapped forms,^115^ and lipidated CMPs.^116^ These studies highlight the stabilizing effect of the *n* → π* interaction and the solvent’s critical role. Additionally, lipidation creates a local hydrophobic environment that accelerates collagen folding, emphasizing the local environment’s influence on peptide folding—a key factor in designing biocompatible materials with improved folding kinetics.

#### How Can We Investigate the Effect of Water on Collagen Assembly and Fibril Properties?

Turbidity and rheology are commonly used methods to investigate the effect of water on the collagen assembly.^27,45,117^ Turbidity provides mostly information about the kinetics of the process, but little insight into the structural properties of the fibril network.^118,119^ By probing the mechanical properties of the network, rheology offers instead more detailed structural information.^120,121^ To study the effect of water on the fibril properties, imaging techniques, such as electron microscopy (including cryo-EM, TEM and SEM), or atomic force microscopy, can be also employed.^45,48,117^ In particular, confocal reflectance microscopy can be performed in an aqueous solution, enabling to monitor the fibril network in collagen native environment and also to follow the network formation during assembly (although only at micrometer resolution due to inherent optical limitations).^120,122^ In addition to these standard microscopy methods, researchers can also use second harmonic generation (SHG)^123^ or Raman microscopy^124^ to investigate the effect of hydration on the structure of the collagen network in *ex vivo* or *in vivo* models.

Sum frequency generation (SFG) spectroscopy has emerged as a powerful tool for investigating protein–water interactions at interfaces, offering unique insights into the molecular-level processes that drive protein assembly and hydration dynamics.^125^ This technique is particularly valuable for studying the behavior of proteins at various interfaces, where the interplay between protein molecules and water plays a crucial role in determining the structure and properties of the resulting assemblies. SFG spectroscopy has been employed to study collagen at interfaces and has enhanced our understanding of its structure and assembly.^126,127^ Notably, examining the polarization-dependent SFG response of collagen has provided detailed insights into its molecular geometry and fibrillar orientation.^128^ SFG spectroscopy can be utilized as a microscopy technique, making it highly effective for studying spatially heterogeneous samples such as collagen fibrils.^129,130^ Moreover, SFG spectroscopy offers real-time, in situ information about the collagen structure and assembly without the exogenous labeling.^131^ In these SFG studies, the primary focus has been on the molecular vibrations of collagen’s aliphatic, carbonyl, and NH groups, while relatively less attention has been given to the vibrations of water molecules in its vicinity. Incorporating information about the hydration water of collagen will enhance the molecular understanding of collagen and may even challenge the current perspective. For example, by providing interface-selective vibrational spectra, SFG spectroscopy has revealed important details about the hydrogen bonding networks and electrostatic interactions that govern protein–water interactions of fused in sarcoma (FUS) protein.^132^ Integrating SFG spectroscopy with complementary computational and experimental methods can provide a comprehensive view of collagen self-assembly at interfaces, offering deeper insights into this complex molecular phenomenon.

Computationally, coarse-grained (CG) models have been developed to provide mechanistic insights into collagen aggregation and the associated thermodynamic and kinetic properties over extended time and length scales. By simplifying molecular representations, coarse-graining captures essential interactions while sacrificing the fast degrees of freedom in a system, thus granting access to experimentally relevant scales. CG models are powerful tools to investigate how different peptide sequences and solvent conditions—parametrized from atomistic simulations—affect collagen fibril assembly, periodicity, and mechanical properties. CG models often employ implicit solvent representations to enhance the simulation efficiency while still capturing the effects of hydration on collagen assembly. Although implicit solvation is a valuable approach, given water’s ubiquitous role in biological systems, some coarse-grained models, especially within the MARTINI force field framework,^133^ have been developed to explicitly represent solvent particles. The MARTINI force field was further extended to include parameters for hydroxyproline (Hyp) and to accurately represent the unique structure of the collagen triple helix, in explicit water, thereby allowing for a precise description of the key mechanical and biophysical properties of collagen molecules.^134^ In summary, coarse-grained models, particularly the MARTINI force field, provide powerful tools for studying collagen aggregation and its mechanical properties over longer time and length scales. Although, to the best of the authors’ knowledge, no MARTINI CG simulations of a full collagen fibril in explicit water have been reported, this approach holds significant potential. Simulating the fibril in explicit water could offer valuable insights into the role of hydration in determining the fibril properties and linking microscopic interactions to macroscopic material characteristics.

At even lower resolution, single particle models can aid in understanding the molecular origin of collagen self-assembly into different morphologies.^45,135,136^ In coarse-grained simulations, the effects of the solvent can be accounted for implicitly in the effective interactions between different molecules and by using methods which allow the fine-tuning of the viscosity of the solvent, e.g., Brownian/Langevin dynamics.^500,501^ Naturally, the pool of techniques extends into continuum models to study tissues, yet these go beyond the scope of this review. We refer the interested reader to a review addressing the importance of continuum models,^137^ yet their potential in terms of collagen hydration effects remains to be explored.

#### How Can We Investigate the Hydration Shell?

To fully understand how water influences collagen structure and self-assembly, it is also crucial to directly study the hydrating water network. Among all the experimental methods, nuclear magnetic resonance (NMR) and IR spectroscopy are among the most suitable. Based on different water dynamics, NMR can distinguish between different types of water molecules. Water molecules that are involved in the formation of the network around collagen show a slower dynamics with respect to the bulk water.^12,14^ This makes it possible to directly disentangle and study the dynamical properties of the hydration shell and its interactions with collagen. Studying water vibrations with IR can also reveal the dynamics and structural heterogeneity of water molecules in fibrils,^138,139^ which are believed to be nanoconfined between the collagen monomers.^11,20^ Combining IR with rheology (rheo-ATR^140,141^) could be a powerful approach for studying the collagen hydration during the self-assembly process, but also during mechanical deformation.

Full-atomistic molecular dynamics (MD) simulations are powerful tools for investigating the hydration shell of biomolecules, such as proteins, DNA, and phospholipids. Various dynamic properties of water in the hydration shell can be analyzed, including rotational and translational dynamics, hydrogen-bond behavior, and residence times (i.e., how long a water molecule stays in the hydration shell before it moves into the bulk). Time-correlation functions (tcfs) are used to study the reorientation of water molecules, providing a site-specific view of water behavior around the biomolecule.^142^ This site-resolved analysis differentiates between hydrophobic regions, hydrogen-bond donors, and acceptors on the biomolecule’s surface, allowing for a comprehensive understanding of water dynamics. The results from these simulations can be correlated with experimentally accessible quantities such as anisotropy decays from ultrafast infrared spectroscopy and orientation relaxation times from magnetic relaxation techniques. Therefore, MD simulations have become essential for exploring hydration dynamics, significantly advancing our understanding of biomolecular stability and function.^138^ In ref (65), MD simulations were used to analyze collagen’s response to changes in its hydration shell. These simulations can capture changes in water organization, such as the formation of monolayers between tropocollagen molecules at low hydration, and can differentiate the responses of specific regions such as the gap and overlap regions. Additionally, MD allows for the analysis of water diffusion within collagen, revealing anisotropic behavior and subdiffusive regimes. Thus, MD provides a detailed, site-specific approach to understanding the hydration shell’s role in the behavior of collagen fibrils.

## Conclusions and Future Perspectives

In this review, we have outlined the essential role that water plays in defining collagen properties, ranging from defining the stability of monomeric structures to guiding fiber assembly and determining the mechanical response of collagen networks in tissue. Water has been shown to have an active involvement at each structural level, giving new significance to its presence in our tissues. In particular, we discussed several studies showing that water is crucial in determining the thermal stability of the triple helix, both as a monomer and as part of the fibril and in guiding the assembly process by mediating collagen–collagen interactions. There seems to be a broad consensus in the literature that water significantly influences the nanomechanical properties of the fibril and, consequently, the overall mechanical properties of the tissue, by acting as a lubricant and facilitating smoother sliding during deformation. We discussed various studies that emphasized the importance of water not only for collagen–collagen interactions but also for interactions between collagen and other molecules, focusing particularly on hydroxypatite crystals in bones. Additionally, we revealed substantial evidence linking changes in hydration to tissue dysfunction and clinical presentation. This suggests that water may be an overlooked factor in current research on diseases associated with collagen defects or production, such as osteogenesis imperfecta. Since collagen competes for the water present in our tissues with other molecules, and water has a profound impact on collagen properties, it is highly probable that water–collagen interactions hold greater significance than previously believed. Recognizing this significance and further investigating water-collagen interactions might becrucial, not only in order to pinpoint critical areas for potentially “correcting” defective collagen but also to find out what are the molecular properties required for developing tailored biomimetic synthetic materials.
